# Emotion Recognition Using PPG Signals of Smartwatch on Purpose of Threat Detection

**DOI:** 10.3390/s25010018

**Published:** 2024-12-24

**Authors:** Gyuwon Hwang, Sohee Yoo, Jaehyun Yoo

**Affiliations:** School of AI Convergence, Sungshin Women’s University, 34 da-gil 2, Bomun-ro, Seongbuk-gu, Seoul 02844, Republic of Korea; 20211421@sungshin.ac.kr (G.H.); 20200867@sungshin.ac.kr (S.Y.)

**Keywords:** threat detection, PPG signal, machine learning, smartwatch

## Abstract

This paper proposes a machine learning approach to detect threats using short-term PPG (photoplethysmogram) signals from a commercial smartwatch. In supervised learning, having accurately annotated training data is essential. However, a key challenge in the threat detection problem is the uncertainty regarding how accurately data labeled as ‘threat’ reflect actual threat responses since participants may react differently to the same experiments. In this paper, Gaussian Mixture Models are learned to remove ambiguously labeled training, and those models are also used to remove ambiguous test data. For the realistic test scenario, PPG measurements are collected from participants playing a horror VR (Virtual Reality) game, and the proposed method validates the superiority of our proposed approach in comparison with other methods. Also, the proposed filtering with GMM improves prediction accuracy by 23% compared to the method that does not incorporate the filtering.

## 1. Introduction

A smartwatch provides rich sensor data that enable the monitoring of individual health conditions. Many commercial smartwatches offer basic health condition insights, such as heart rate and stress level, based on bio-signal measurements. The most common sensors used are the ECG (electrocardiogram) and PPG (photoplethysmogram). Most smart watches also require users to make contact with the watch with the contralateral hand/finger in order to measure with the ECG. However, PPG signals can often be collected without specific requirements. Numerous studies have been conducted to infer health conditions using ECG and PPG data [1,2,3,4,5,6,7,8].

The background of this research comes from the development of policing technology aimed at protecting individuals. For those who wear a smartwatch, the system aims to automatically report an SOS event when the wearer faces a threat. When a wearer feels a threat, it causes a change in the PPG signal pattern. The required technology is to detect the changed pattern as quickly as possible for the purpose of emergency rescue. Thus, the objective of our research is to develop an algorithm to determine if a smartwatch wearer is experiencing fear using only the short-term PPG signal.

However, due to the low-cost sensors used in commercial smartwatches, the signals they generate are not only low frequency but also prone to noise, which makes them easily affected by motion artifacts and external factors [9,10]. This sets our work apart from other emotion detection studies that rely on expensive sensor devices.

Data integrity is a critical issue when collecting training data from participants for threat detection problems. In fear-inducing experiments, individual responses vary significantly; some participants may experience intense fear, while others remain unaffected. This variability leads to inaccurate data labeling and hinders the development of effective detection models.

The main contribution of this paper is the development of a machine learning method to detect threats using short-term signals from a commercial smartwatch. In order to address data integrity, a filtering algorithm is proposed to identify and utilize only the most relevant training data, ensuring the creation of a robust threat detection model. We employ a GMM (Gaussian Mixture Model) [11] to differentiate between normal and anomalous data points. For the prediction model, we utilize a 1D-CNN (one dimensional Convolutional Neural Network) to detect threats effectively.

Experiments conducted with participants engaging in a horror VR (Virtual Reality) game demonstrate that our proposed GMM-based filtering improves threat detection accuracy by 16% compared to the method, which does not apply the GMM but retains the same backbone network. Furthermore, comparisons with some other emotion detection algorithms validate the superiority of our proposed approach.

The rest of this paper is as follows. Section 2 describes a preprocessing method to convert raw PPG signals to trainable data. Section 3 and Section 4 remark the proposed filtering algorithm and the prediction model, respectively. Section 5 analyzes the experimental results, and Section 6 and Section 7 summarize the discussion and conclusion, respectively.

## 2. Preprocessing

**Figure 1:**
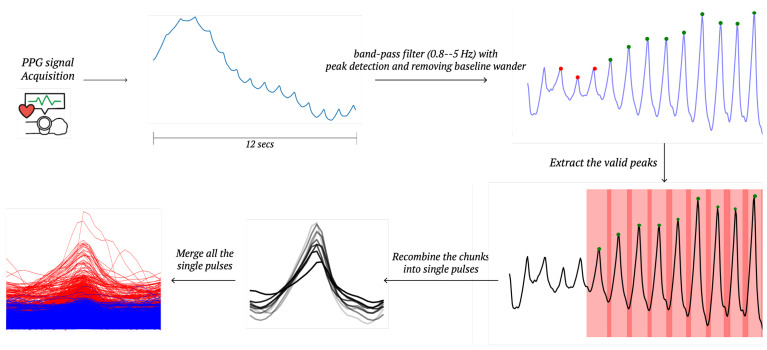
During the preprocessing stage, single pulses are extracted through filtering from the raw PPG signals collected by a smartwatch.

In this paper, 25 Hz green light PPG signals are utilized. These signals are measured using a smartwatch (Samgsung Galaxy Watch 5, Samgsung, Suwon, Republic of Korea) with the help of the Samsung Health SDK through an open Python library. The signal readings span a 12 s period, so the algorithm starts with 25×12=300 number of time-series measurements. The primary goal of the preprocessing stage is to transform the 12 s time-series data into single pulses while filtering out any poor-quality pulses as depicted in Figure 1. From the 12 s data containing 300 samples, some single pulses are extracted, with each pulse covering 1.1 s and consisting of 27 samples.

The 12 s data are first passed through a bandpass filter with cutoff frequencies ranging from 0.5 Hz to 8 Hz. This frequency range is selected based on experimental findings, as frequencies above 8 Hz typically represent high-frequency noise, while those below 0.5 Hz are considered baseline fluctuations in the PPG signals. The next step involves identifying valid peaks. Excluding false peaks is a common method for feature extraction from PPG signals, particularly to mitigate the impact of motion artifacts and other noise sources [12,13]. Single pulses are then generated, with each pulse centered around a valid peak, including 13 samples (0.52 s) before the peak and 14 samples (0.56 s) after it. Finally, if a single pulse exceeds a specified threshold, which is set to 25,000 in this paper, it is removed ensuring outlier removal.

The process of the filtering and peak detection from reading the raw PPG data to obtaining single pulses is a well-established method [14]. This approach can be effectively implemented in several open-source libraries. In this paper, the Heartpy library [15,16] is utilized to carry out the bandpass filter and to detect valid peaks.

## 3. GMM Filtering

When collecting raw PPG data from participants, the signals are labeled as either 1 or 0. PPG data recorded while watching fear-inducing videos, such as those depicting accidents or acts of terrorism, are labeled as 1. In contrast, data gathered while watching calming videos, such as those featuring baby animals or serene natural scenes, are labeled as 0.

However, data obtained from psychological experiments can have a significant drawback such that the annotations might be inaccurate. Some participants may feel fine even when watching unpleasant clips, while others might feel distressed during calming videos. Based on our experience, using such naively labeled training data leads to poor performance in classification by any typical machine learning algorithm.

**Figure 2:**
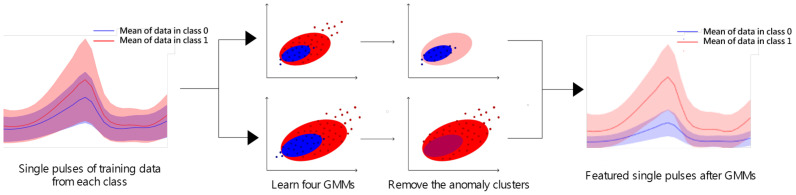
During the filtering stage, some data are selected from the original single-pulse data using the GMM method. Many of the original heart beats belong to distributions (shaded color) of 0 and 1 classes. However, after applying GMM, the new dataset of heart beats becomes more clearly distinguishable between the two classes.

To address this problem, we apply the GMM (Gaussian Mixture Model) to select only the heart beats that are clearly distinguishable between two classes. Figure 2 shows how GMM is used for filtering among the two class data points. Without the application of GMM, the original data points overlap in their distribution, regardless of the class, making it difficult for each class data point to represent its identity accurately. GMM is used to eliminate these ambiguously overlapping data points, retaining only the trainable sets. This post-processing step with GMM significantly improves the learning performance in our experiments. Since GMM is one of the most famous machine learning algorithms, this paper omits its mathematical definition and optimization process. For those details, please refer to [11]. Instead, we will describe our specific problem formulation for applying GMM in the following.

We assume that each data point belonging to a class can be categorized as either normal or anomalous. For example, if the data collected from a displeased participant exhibit negative features, they are considered normal; otherwise, they are classified as anomalous. To incorporate this definition into the GMM formulation, we assume that both normal and anomalous data follow their own Gaussian distributions. The mean and variance of each GMMs are learned through an optimization process. In summary, two Gaussian models are trained: one representing the normal and anomalous distributions of the 1 class, and the other representing the 0 class. As a result, all data points including training and test data, which pass through the preprocessing step in Section 2, become the input of two GMMs. When both models predict abnormal for a data point, it is treated (or removed) as an indiscernible single pulse.

**Figure 3:**
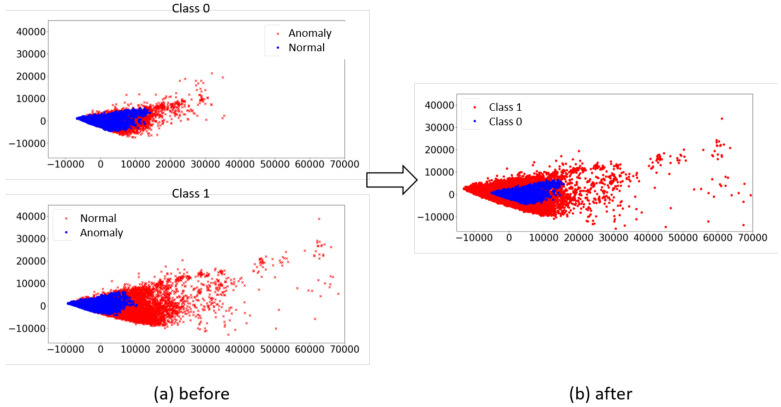
The impact of the GMM as filtering is illustrated using actual single pulse data. In (**a**), two GMM models, one for each 1 and 0 class, categorize their respective data into normal and anomaly groups. In (**b**), only the data within the normal groups from each class are chosen as training data. This process eliminates ambiguously labeled data that may have incorrect ground truth, significantly enhancing the learning performance for threat detection.

Figure 3 illustrates the impact of GMM on the actual data. Since each PPG single pulse is 27-dimensional, we reduce the dimensionality to two for visualization purposes using PCA (Principal Component Analysis) [17]. In Figure 3a, the four types of data resulting from the GMM learning process are displayed. Within both classes 1 and 0, the anomalous and normal data are intermixed. However, in Figure 3b, the clearly distinguishable data are organized by selecting only the normal data from both the 1 and 0 classes shown in Figure 3a. These selected data are then exclusively used for training, while the others are removed from the training dataset.

## 4. Prediction Model

One-dimensional CNNs are effective for time-series forecasting and can be applied to biometric data like PPG signals [18,19,20]. This architecture features multiple layers that each contribute to feature extraction and classification. It takes 27-dimensional inputs corresponding to the size of a single pulse and produces binary outputs. The cross-entropy loss function, Adam optimizer, early stopping at the 30th epoch, and a 20% validation set are used for training. The proposed 1D-CNN architecture used for the prediction of the binary class is detailed in Table 1.

In our experience, alternative prediction models might offer better performance in another application. This paper primarily focuses on validating the effects of the filtering, and while 1D-CNNs are effective in certain contexts, they may not be the best solution for every application, such as when dealing with other physiological sensors such as an electrocardiogram sensor.

Finally, the determination of whether a threat is identified relies on a rule. It is important to note that the input to the 1D-CNN consists of a 27-dimensional single pulse spanning 1.1 s, while the prediction period covers 12 s. The number of single pulses within a 12 s dataset varies depending on the filtering effects by both preprocessing and GMM. In out setup, ‘threat’ is identified if 33 % or more of the results from the 1D-CNN are ‘1’ in 12 s, or conversely, if this threshold is not met.

## 5. Methods and Experiments

### 5.1. Compared Methods

We applied four different models to address the same threat detection problem. The algorithms were selected based on a standard applicable to smartwatches, which allows for the analysis of 12 s of PPG time-series data. For instance, we excluded methods that utilize features extracted from the frequency domain, as such features typically require measurement periods of at least 90 s [21,22].

The first architecture of the comparison model, as reported in [23,24], combines CNN and LSTM networks to capture both spatial and temporal features from the PPG signal. In this model, preprocessing involves segmenting the PPG data and normalizing them to remove the baseline noise. The segmented 4-second PPG data are first fed into a CNN to extract spatial features, which are then passed to an LSTM to capture temporal dependencies. This combined architecture leverages CNNs for feature extraction and LSTMs for sequential data processing. Since the network processes 4-second data segments, a threat is determined if at least one of the outputs from three consecutive network evaluations is negative.

The second model architecture, as reported in [25,26], incorporates an additional feature known as the NN-interval, which is extracted from a 10 s PPG signal. The NN-interval refers to the time interval between consecutive normal R-peaks. By using this feature within a 1D-CNN, the model aims to filter out artifacts caused by arrhythmic events or faulty sensors. Since the input consists of 10-second data segments, the threat is determined directly by the network’s output. The compared existing models were fine-tuned to yield the best performance results. For example, we tuned the cutoff frequency in the filtering step and some hyper-parameters of the prediction models.

The third model, which does not use the GMM but retains the same backbone network as ours, takes a single pulse as the input. Since the GMM is not applied in this method, the amount of training data used differs from that in the proposed method.

### 5.2. Training Data

To gather training data, we recorded PPG signals from 20 participants. They watched a total of 74 min and 14 s of peaceful videos and 66 min and 29 s of offensive videos. The PPG signals were collected from the participants’ wrists using a Galaxy Watch 5.

The methods being compared differ in the amount of training data used, due to variations in the preprocessing and input size. The first model is trained on 21,910 data points, with 12,199 from class 0 and 9711 from class 1, and an input size of 100. The second model uses 8619 data points, including 4807 from class 0 and 3812 from class 1, with an input size of 250. The third model, which does not utilize GMM filtering, is trained on 161,268 data points, including 87,505 from class 0 and 73,763 from class 1, with each data point representing a single pulse and an input size of 27. In contrast, our model, which applies GMM filtering, is trained on 83,051 data points, consisting of 43,945 from class 0 and 39,106 from class 1, with an input size of 27.

### 5.3. Result of Test #1

We conducted the first simulation for the test using 10% of the data, which were split from the training set. The results are shown in Table 2. Models 1 and 2 achieved high precision but low recall, indicating a tendency to classify threats as normal. Model 3 exhibited the opposite behavior. In contrast, our method, which incorporates GMM, produced balanced predictions without bias, resulting in superior performance compared to the other models.

### 5.4. Result of Test #2

For another test scenario, PPG signals were measured from 8 volunteers wearing identical smartwatches on their wrist under two conditions: resting, which represents 0 class, and playing a horror game, which represents 1 class. During the resting condition, each participant was instructed to remain calm and stationary for a few minutes to allow measurement of the baseline trends of the PPG signal.

For the 1 class test, the participants played the horror Virtual Reality (VR) game “Resident Evil 7”. The game scenario was consistent for all participants, with a defined beginning and end, though playtime varied slightly depending on the individual skill in VR gaming. Data collected from scenes following a horrific event in the game, lasting approximately 5 min, were specifically annotated as 1. Data from other parts of the game, in which players usually move and find items, were not used for testing.

Table 3 summarizes the results from eight different tests. All the compared methods have an average accuracy of less than 50%, as they tend to be biased toward predicting a specific class, similar to the simulation results in Section 5.3. In contrast, our model achieves the best performance.

**Figure 4:**
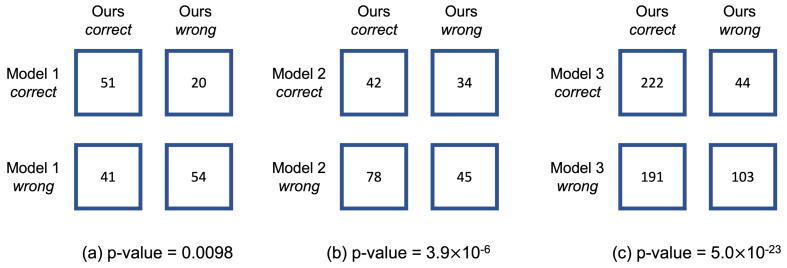
McNemar’s test for classifier comparisons: (**a**) between Model 1 and our model, (**b**) between Model 2 and our model, and (**c**) between Model 3 and our model.

Additionally, we performed McNemar’s test for three comparisons: one between Model 1 and our model, two between Model 2 and our model, and three between Model 3 and our model. Since the compared models use different numbers of test data points due to varying preprocessing methods, we adjusted the test data points to match the model that uses the fewest. Figure 4 presents the results of McNemar’s test for classifier comparisons. All comparisons yield p-values less than 0.01.

## 6. Discussion

There is a most common method to categorize emotions, called the valence and arousal problem [27]. According to this theory, emotions are mapped within a two-dimensional (2D) space based on their valence and arousal levels. Valence, typically represented on the horizontal axis, indicates the level of pleasantness, while arousal, often shown on the vertical axis, reflects the degree of activation. For example, anger is characterized by low valence and high arousal, while joy is associated with high valence and high arousal. Therefore, identifying an emotion involves two binary classifications: determining whether valence and arousal are high or low.

In this paper, PPG data are collected in two specific scenarios: watching peaceful clips, representing class 0, and viewing offensive clips, representing class 1. This approach assumes that the pattern of the PPG signal during a threatening situation would resemble that of offensive clips, as it is not feasible to collect data from real-life threatening situations. Hence, the developed model may also respond similarly to other emotions, such as excitement, which could lead to unintended classifications. In future work, it will be important to analyze which emotions trigger responses in the developed model and explore ways to filter out non-threat-related emotions, focusing specifically on threat detection.

This research is part of a broader project focused on threat detection using smartwatches, specifically exploring emotional anomaly detection, in addition to physical and geographical anomaly detection. The main challenge in predicting emotions using bio-sensors, such as through PPG signals, is that physical movement can introduce noise, causing fluctuations in PPG readings that may be mistaken for emotional changes. To address this, another side of research proposes using Inertial Measurement Units (IMUs), including accelerometers and gyroscopes, to monitor movement. This additional data help differentiate between actual emotional changes and movement-induced artifacts in the PPG signal, preventing false alarms in emotion prediction models. A key aspect of this research is to validate the algorithm in real-world scenarios. This will involve identifying any issues that arise when the system is deployed in practical settings. The ultimate aim is to demonstrate the effectiveness of the algorithm through a demonstration with a police agency, providing a real-world proof of concept.

## 7. Conclusions

This paper addressed the problem of threat detection using short-term PPG signals collected from a smartwatch. The experimental results confirmed the effectiveness of the proposed filtering method, demonstrating improved accuracy compared to the methods that did not incorporate GMM. For future work, we plan to explore ways to surpass the current best accuracy of 72%, potentially by developing a more robust prediction model, such as applying a transformer architecture.

## Figures and Tables

**Table 1:** The 1D-CNN architecture for binary classification with a single pulse input.

Layer (Type)	Input Shape	Output Shape
Input (Input Layer)	(None, 27, 1)	(None, 27, 32)
conv1d 0 (Conv1D)	(None, 25, 32)	(None, 25, 32)
conv1d 1 (Conv1D)	(None, 25, 32)	(None, 25, 32)
max pooling1d 0 (MaxPooling1D)	(None, 25, 32)	(None, 11, 32)
conv1d 2 (Conv1D)	(None, 11, 32)	(None, 9, 32)
conv1d 3 (Conv1D)	(None, 9, 32)	(None, 7, 32)
max pooling1d 1 (MaxPooling1D)	(None, 7, 32)	(None, 3, 32)
conv1d 4 (Conv1D)	(None, 3, 64)	(None, 1, 64)
flatten (Flatten)	(None, 1, 64)	(None, 64)
dense 1 (Dense)	(None, 64)	(None, 8)
dropout 1 (Dropout)	(None, 8)	(None, 8)
dense 2 (Dense)	(None, 8)	(None, 4)
dropout 2 (Dropout)	(None, 4)	(None, 4)
dense 3 (Dense)	(None, 4)	(None, 1)
dropout 3 (Dropout)	(None, 1)	(None, 1)

**Table 2:** Simulation results of threat detection using watching-clip data.

Metric	Model 1	Model 2	Model 3	Ours
Accuracy	0.6329	0.7109	0.6109	0.8631
F1 score	0.5653	0.5653	0.4756	0.8611
Recall	0.4190	0.2090	0.8250	0.9967
Precision	0.8690	0.8690	0.3343	0.7581

**Table 3:** Experimental results of threat detection from 8 different tests. All the participants in the experiment are women in their twenties.

	Metric	Model 1	Model 2	Model 3	Ours
Subject 1	Accuracy	0.5529	0.4059	0.7283	0.7037
	F1 score	0.7121	0.329	0.7441	0.800
	Recall	0.8393	0.209	0.9696	0.7164
	Precision	0.6184	0.778	0.6037	0.9056
Subject 2	Accuracy	0.3253	0.3711	0.3116	0.4285
	F1 score	0.4717	0.179	0.0701	0.3714
	Recall	0.463	0.109	0.400	0.7222
	Precision	0.4808	0.500	0.0384	0.25
Subject 3	Accuracy	0.5135	0.4023	0.4929	0.7605
	F1 score	0.6786	0.036	0.3333	0.7901
	Recall	0.8444	0.019	0.900	0.8648
	Precision	0.5672	0.500	0.2045	0.7272
Subject 4	Accuracy	0.5972	0.4235	0.5178	0.8214
	F1 score	0.7478	0.036	0.5263	0.8717
	Recall	1.000	0.020	1.000	0.9444
	Precision	0.5972	0.200	0.3571	0.8095
Subject 5	Accuracy	0.4576	0.5493	0.5357	0.7857
	F1 score	0.6279	0.216	0.1875	0.7391
	Recall	0.9643	0.121	0.500	0.850
	Precision	0.4655	1.000	0.1153	0.6538
Subject 6	Accuracy	0.6053	0.4667	0.3783	0.9054
	F1 score	0.7541	0.133	0.3030	0.9247
	Recall	0.9787	0.071	0.500	0.9148
	Precision	0.6133	1.000	0.2173	0.9347
Subject 7	Accuracy	0.6282	0.3696	0.3947	0.6842
	F1 score	0.7717	0.000	0.1153	0.700
	Recall	1.000	0.000	0.750	0.875
	Precision	0.6282	0.000	0.0625	0.5833
Subject 8	Accuracy	0.0303	0.443	0.4843	0.6718
	F1 score	0.0588	0.043	0.1538	0.6037
	Recall	0.0541	0.022	1.000	0.9411
	Precision	0.0645	1.000	0.0833	0.4444
Total	Accuracy average	0.4638	0.4289	0.4804	0.7201

## Data Availability

The data presented in this study are available upon reasonable request made to the corresponding author. The data are not publicly available due to privacy restrictions.
